# The dual regulatory role of METTL14-mediated m^6^A modification in tumorigenesis and its underlying mechanisms

**DOI:** 10.3389/fonc.2026.1771313

**Published:** 2026-03-04

**Authors:** Yulan Yang, Lemuge Chao, Xudong Ao, Junqing Liang

**Affiliations:** Peking University Cancer Hospital/Affiliated Cancer Hospital of Inner Mongolia Medical University, Inner Mongolia Key Laboratory of Cancer Intersection and Innovation, Hohhot, Inner Mongolia, China

**Keywords:** m6A modification, METTL14, non-coding RNA, targeted therapy, tumor microenvironment

## Abstract

N^6^-methyladenosine (m^6^A), as the most abundant RNA epitranscriptional modification in eukaryotes, its key component of the methyltransferase complex, METTL14, not only cooperates in catalyzing m^6^A deposition but also has functions independent of methyltransferase activity. This article systematically reviews the dual regulatory role of METTL14 in tumors and its molecular mechanisms, mainly organizing the relevant research in a logical sequence of “tumor suppressive effect - tumor promoting effect - controversial or context-dependent”. Studies have shown that METTL14 often plays a tumor suppressive role in tumors such as hepatocellular carcinoma and colorectal cancer, while in pancreatic cancer and nasopharyngeal carcinoma, it mostly promotes malignant progression, showing a high degree of context dependence. This article focuses on two key mechanisms: on the one hand, METTL14 precisely regulates the processing, stability, and function of non-coding RNAs (including miRNAs, lncRNAs, and circRNAs) through m^6^A modification, reshaping the competitive endogenous RNA (ceRNA) network; on the other hand, it shapes an immunosuppressive tumor microenvironment by directly upregulating immune checkpoints such as PD-L1, mediating metabolism-immune interactions, and regulating the function of immune cells. Its functional duality also stems from the selective regulation of key pathways such as PI3K/AKT, as well as the differential interpretation by different m^6^A readers (such as YTHDF2 and IGF2BPs). Given the close association of these mechanisms with clinical prognosis, the expression level of METTL14 shows significant potential as a prognostic marker and therapeutic target; in the future, it is necessary to combine single-cell multi-omics and other technologies to analyze its dynamic regulatory network in specific tumor contexts and explore precise treatment strategies based on synthetic lethality or targeting downstream effector molecules.

## 1 Introduction

N^6^-methyladenosine (m^6^A) is one of the most abundant and well-studied epitranscriptomic modifications in eukaryotic messenger RNA (mRNA), playing a central role in gene expression regulation by precisely controlling RNA splicing, translation, stability, and degradation. This dynamic and reversible modification system is regulated by a trio of methyltransferases (“writers”, such as the METTL3-METTL14 complex), demethylases (“erasers”, such as FTO and ALKBH5), and recognition proteins (“readers”, such as the YTHDF family and IGF2BPs), and its dysregulation is closely associated with the occurrence and development of various diseases, including cancer (1).

In the m^6^A methyltransferase complex (MTC), METTL14 not only serves as the structural core for forming a stable heterodimer with METTL3, ensuring the correct assembly and catalytic function of the complex, but recent studies have also revealed its multiple functional dimensions independent of the classical m^6^A writing activity. It can act as a METTL3-independent chromatin regulator to influence transcription (2), play a key role in specific cell fate decisions (such as reprogramming) (3), and endow the entire complex with target specificity due to its unique substrate recognition module (such as preference for RNA G-quadruplexes) (4, 5). This “cooperative and independent coexistence” characteristic forms the basis for understanding the functional complexity of METTL14.

In the field of tumor biology, METTL14 has attracted significant attention due to its extensive regulatory network and exhibits a highly context-dependent dual function: it often acts as a tumor suppressor in liver cell carcinoma, colorectal cancer, and other tumors (6, 7), while in pancreatic cancer, nasopharyngeal carcinoma, and other types, it often promotes malignant progression (8). This “duality” stems from the vast differences in the downstream target networks it regulates through m^6^A modification. Its molecular mechanism is extremely complex, encompassing precise programming of the life cycle and function of non-coding RNAs (miRNA, lncRNA, circRNA) (such as regulating pri-miRNA processing through DGCR8, bidirectional regulation of lncRNA/circRNA stability through different readers, and reshaping the competitive endogenous RNA (ceRNA) network), regulation of the translation and stability of key signaling pathway node mRNAs, and profound impacts on tumor metabolic reprogramming and the immune microenvironment (9–14). Additionally, the expression level of METTL14 is significantly correlated with tumor stage, metastatic potential, and patient prognosis, suggesting its potential as a prognostic marker and therapeutic target, such as in reversing chemotherapy resistance or modulating the response to immunotherapy (15–17). However, the intrinsic mechanisms underlying its functional differences and its overall regulatory network remain incompletely elucidated, urgently requiring systematic mechanistic analysis and translational research.

**Figure 1:**
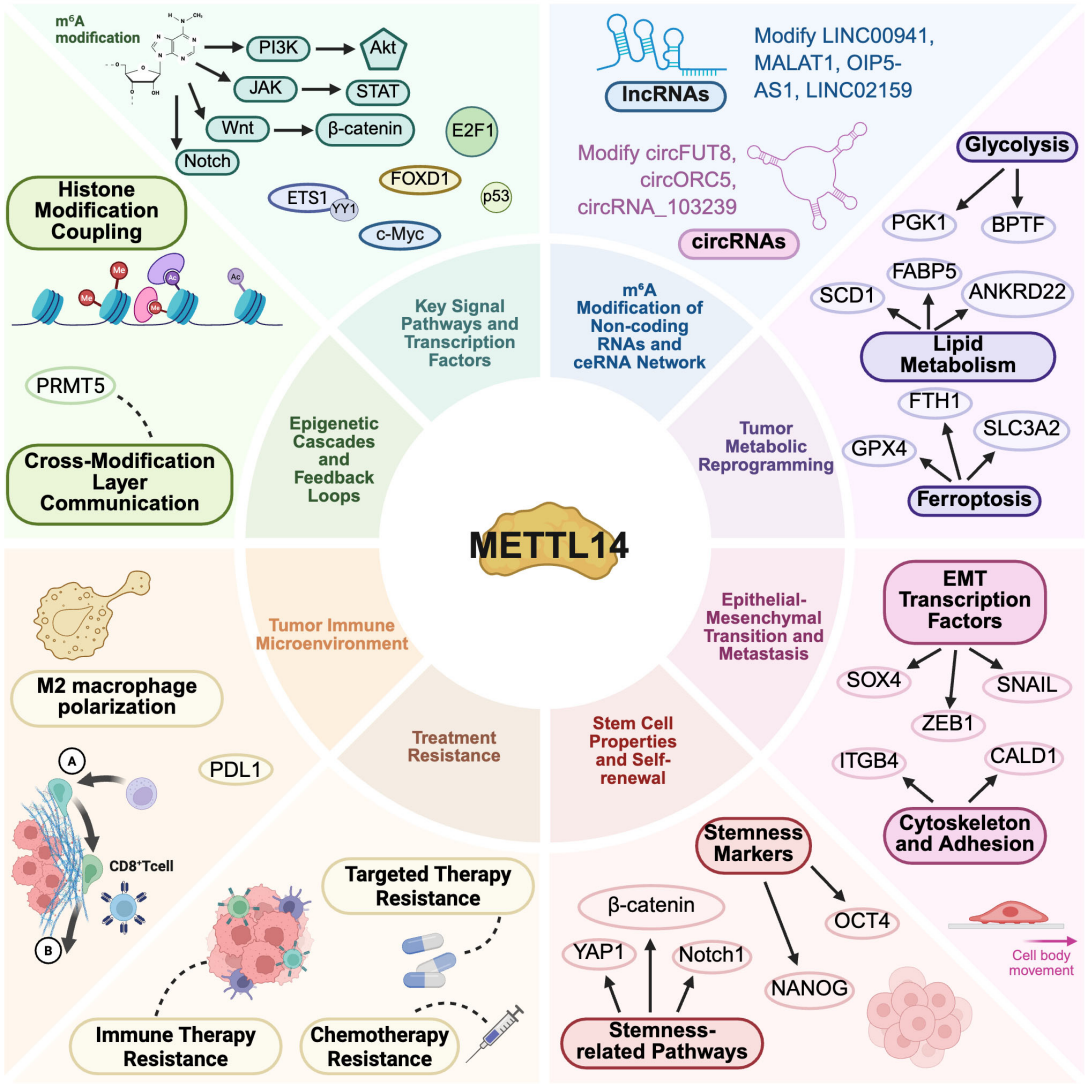
Core regulatory mechanisms of METTL14 in cancer: This figure is divided into multiple sections, encompassing key signaling pathways, histone modifications, non-coding RNA networks, tumor metabolism, immune microenvironment, therapeutic resistance, stemness, epithelial-mesenchymal transition (EMT), and cytoskeletal regulation. Each section details the specific pathways, molecules, and biological processes involved.

In summary, METTL14, as the structural and functional core of the m^6^A methylation apparatus, is widely involved in the malignant progression of tumors. As shown in **Figure 1**, its regulatory network mediated by m^6^A modification can be systematically summarized into several key aspects: regulation of key signaling pathways and transcription factors; reshaping the gene regulatory network through non-coding RNAs (miRNA, lncRNA, circRNA); driving metabolic reprogramming, epithelial-mesenchymal transition, and metastasis; maintaining the characteristics of tumor stem cells; mediating treatment resistance; shaping an immunosuppressive microenvironment; and interacting with other epigenetic modifications. In-depth analysis of this network will not only reveal new dimensions of tumor progression but also provide important theoretical basis and potential new targets for achieving precise diagnosis and treatment.

## 2 Structural basis and functional independence of METTL14

### 2.1 Structural features and catalytic functions of METTL14

METTL14 serves as the structural and functional core of the m^6^A methyltransferase complex (MTC). Its protein architecture is centered on a methyltransferase (MTase) domain, which harbors a conserved S-adenosylmethionine (SAM)-binding motif that is indispensable for methyl transfer reactions. Critically, METTL14 acts as a key scaffold protein to form a stable heterodimer with the catalytic subunit METTL3—a prerequisite for maintaining the MTC’s conformational integrity, subcellular localization, and recruitment of regulatory cofactors such as WTAP (18–20). This scaffolding role directly dictates the intracellular abundance of functional MTC, thereby exerting global control over m^6^A modification levels; dysregulation of global m^6^A is a hallmark of numerous tumor types.

Substrate recognition by METTL14 is a cornerstone of its context-dependent functionality. Its N-terminal domain mediates direct binding to target RNAs, while symmetric dimethylation of arginine residues within the RGG/RG-rich region (shared with METTL3) substantially enhances the complex’s RNA-binding affinity and substrate specificity (5, 21–23). This enables the METTL3/METTL14 complex to selectively target RNA subsets tightly linked to tumor progression—including transcripts of oncogenes and tumor suppressors—thereby regulating their fate via m^6^A modification. This provides a fundamental structural rationale for METTL14’s dual regulatory roles across distinct tumor contexts.

METTL14’s structure and function are governed by dynamic post-translational modifications (PTMs). For instance, METTL3 competitively binds to METTL14 to shield it from ubiquitin-mediated degradation by the E3 ligase STUB1, stabilizing METTL14 protein levels to sustain m^6^A modifications required for tumorigenesis (24). Additionally, the METTL3/METTL14 complex undergoes liquid-liquid phase separation (LLPS) in the nucleus, a process modulated by SAM concentration and tumor-associated mutations (25). This LLPS capacity allows the complex to dynamically assemble into biomolecular condensates, which efficiently enrich and methylate specific RNA substrates. In response to tumor microenvironmental cues (e.g., metabolic stress), this phase-separated assembly rapidly remodels the cellular epitranscriptome, driving malignant phenotypes such as tumor adaptation and drug resistance.

### 2.2 Independence of METTL14 function: synergy and specific mechanisms with METTL3

METTL14 and METTL3 synergize within the core heterodimer to mediate the majority of m^6^A modifications. However, in-depth investigations have revealed that METTL14’s functions are not entirely dependent on METTL3—instead, it harbors unique regulatory potential independent of its m^6^A methyltransferase activity (2, 3, 26). This forms the structural and mechanistic basis for its context-specific regulatory roles across diverse biological processes.

METTL14 can function independently as an m^6^A-independent chromatin and transcriptional regulator. Its protein architecture encompasses functional modules beyond canonical methyltransferase activity. Dou et al. definitively demonstrated that METTL14 acts as a chromatin regulator independent of its RNA m^6^A methyltransferase activity (2). In mouse embryonic stem cells (mESCs), METTL14 colocalizes with the repressive histone mark H3K27me3, directly binds to this mark, and recruits the histone demethylase KDM6B to erase H3K27me3—thereby actively remodeling chromatin states and promoting gene expression. Notably, this function is entirely independent of METTL3: METTL3 depletion leads to transcriptional upregulation, whereas METTL14 depletion causes global gene repression, indicating opposing roles in core transcriptional regulatory networks (2). Similarly, during cellular senescence, METTL3 and METTL14 undergo genome-wide redistribution: METTL14 is recruited to enhancer regions, while METTL3 localizes to pre-existing NF-κB sites at promoters. Together, they transcriptionally drive the expression of senescence-associated secretory phenotype (SASP) genes—a process shown to be independent of m^6^A mRNA modification (26). These findings reveal that METTL14 can regulate gene expression independently of the m^6^A-writing cycle via direct interactions with chromatin and the transcriptional machinery.

METTL14 exerts independent, critical regulatory roles in specific cell fate decisions. During the precise process of somatic cell reprogramming to induced pluripotent stem cells, overexpressed METTL14 significantly enhances reprogramming efficiency—a function confirmed to be m^6^A-independent (3). Mechanistically, METTL14 cooperates with classical reprogramming factors (e.g., Oct4, Sox2) to transiently activate SASP gene expression specifically in non-reprogramming cell populations. Key SASP components (e.g., IL-6) then act as paracrine signals to promote reprogramming (3). This paradigm reveals that METTL14 can independently sense and integrate reprogramming stress signals, regulating cell population fate via specific secretory phenotypes—with its functional axis decoupled from METTL3’s catalytic activity.

In tumor biology, METTL14 and METTL3 exhibit distinct regulatory networks and functions. An in-depth analysis of esophageal squamous cell carcinoma (ESCC) revealed clear differences in their expression patterns, functional contributions, and correlations with tumor burden (27). Functional assays showed that METTL3 knockdown strongly inhibits cancer cell proliferation *in vitro* and *in vivo*, whereas METTL14 knockdown has a weaker inhibitory effect and does not alter METTL3 protein levels—indicating differential contributions to maintaining cancer cell proliferation (27). Genomic evidence further supports their independence: mRNA sequencing identified 1,615 genes regulated independently by METTL3, compared to only 776 genes co-regulated with METTL14—demonstrating largely non-overlapping target gene networks and downstream signaling pathways (27). Multi-omics analyses also suggested that METTL3 can independently interact with Nop56p-associated pre-rRNA complexes and mRNA splicing machinery, functions potentially independent of METTL14 (27). These findings reveal that while METTL14 and METTL3 act as a complex in cancer cells, their functions have undergone significant decoupling and independent specialization.

METTL14 mediates unique substrate recognition and binding via its specific domains—the physical basis for its functional independence. The RGG repeat sequence in METTL14 is a key domain for specific RNA binding: studies confirmed that this domain enables METTL14 to selectively recognize and bind RNA G-quadruplexes (rG4s), a non-canonical higher-order RNA structure (4). This binding preference directs the METTL3/METTL14 complex to preferentially methylate adenines adjacent to rG4 structures, with substrate selectivity primarily mediated by METTL14 (4). Another study using RNA capture technology further confirmed that endogenous RNAs bound by the METTL3/14 complex are enriched in (GGA) repeats that form rG4s (5). These observations indicate that METTL14, via its unique RNA-binding module, independently determines the complex’s targeting of structured RNA substrates—potentially influencing biological processes including tumorigenesis and progression.

**Figure 2:**
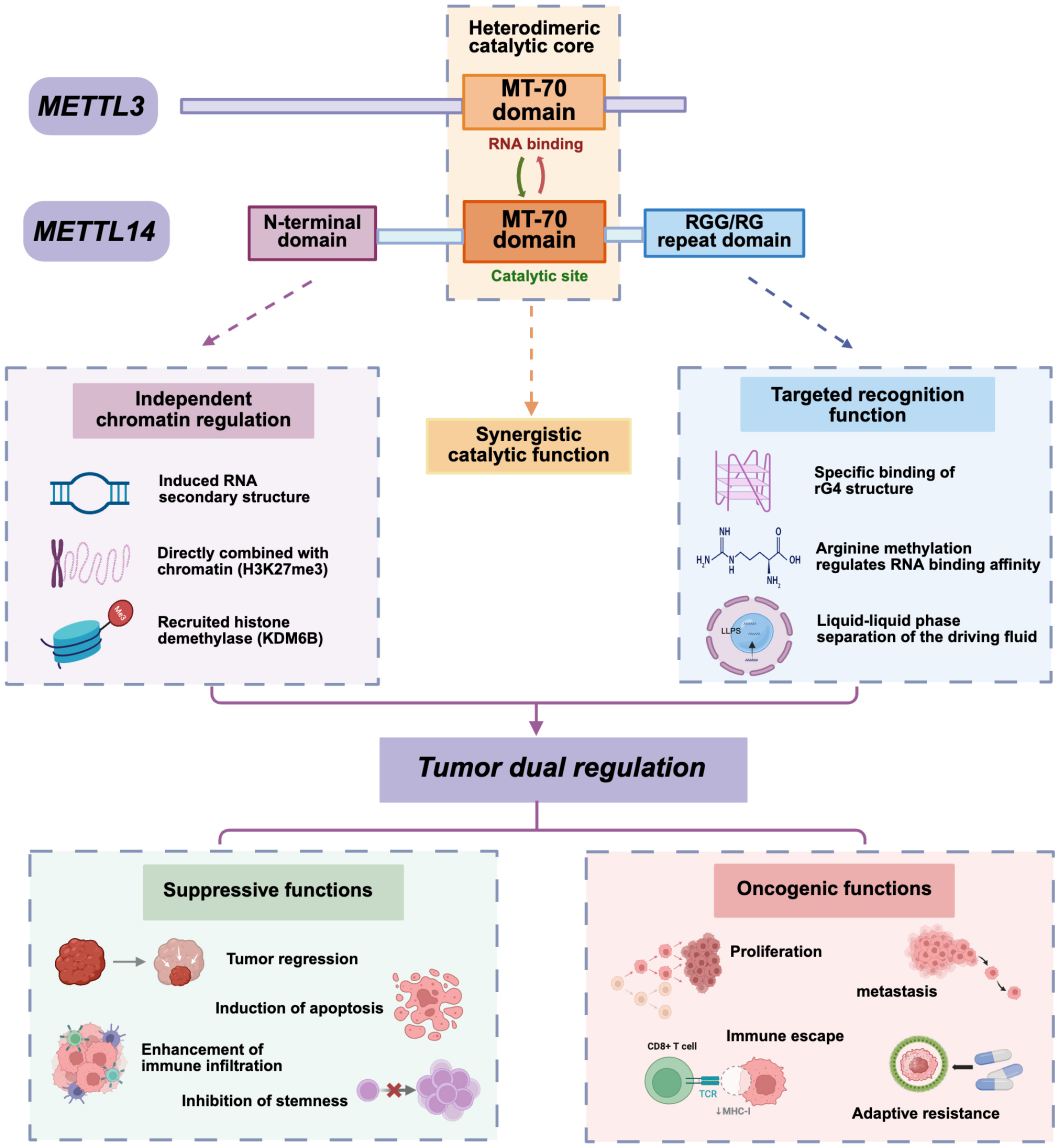
Dual regulatory roles of the METTL3/14 heterodimer: The upper panel illustrates the independent chromatin regulatory domain, the MT-70 domain (responsible for RNA binding and catalytic activity), and the RGG/RG repeat domain. These structural features collectively confer dual regulatory functions on METTL14 in tumorigenesis. The lower panel outlines its inhibitory functions (e.g., tumor regression and immune cell infiltration) and oncogenic functions (including cell proliferation, metastasis, and immune evasion).

In summary, as illustrated in **Figure 2**, METTL14 is not a static catalytic component but a dynamic functional integration platform anchored in its unique domains (N-terminal chromatin regulatory domain, MT-A70 catalytic domain, and C-terminal rG4 recognition domain). Its scaffolding function, substrate-specific recognition capacity, precisely regulated structural plasticity, and specific molecular interactions collectively dictate the targeting specificity and efficiency of m^6^A deposition—while also enabling m^6^A-independent chromatin and transcriptional regulation. These properties directly underpin its ability to modulate oncogenic signaling pathways and cell fate via modification of key cancer-associated RNAs, ultimately manifesting as context-dependent dual roles (tumor-suppressive or oncogenic) and a functional relationship with METTL3 characterized by both cooperation and independence. Deciphering this structure-function interplay is central to unraveling METTL14’s functional diversity and context dependence and serves as the logical foundation for rational design of anti-tumor therapeutics targeting the METTL3-METTL14 complex.

## 3 The dual regulatory role of METTL14 across tumor types and its underlying molecular mechanisms

The functional role of METTL14 in tumor initiation and progression exhibits striking context-dependent duality. As summarized in **Table 1**, METTL14 typically acts as a tumor suppressor (with downregulated expression) in hepatocellular carcinoma (HCC) and colorectal cancer (CRC) (6, 7), while it functions as an oncogene (with upregulated expression) in pancreatic ductal adenocarcinoma (PDAC) and nasopharyngeal carcinoma (NPC) (8, 51). In non-small cell lung cancer (NSCLC) and breast cancer, its role remains controversial or is tightly linked to specific molecular contexts (e.g., genetic subtypes) (9, 71, 85, 86). This functional heterogeneity arises from the substrate specificity of METTL14-mediated m^6^A modification: depending on whether it stabilizes oncogenic transcripts, accelerates tumor suppressor mRNA degradation, activates or represses non-coding RNA (ncRNA) functions, METTL14 selectively modulates key signaling pathways such as PI3K/AKT (33, 40, 62). Critically, this substrate selectivity is collectively shaped by context-specific factors including tumor tissue origin, genetic background (e.g., p53 mutational status) (97), and tumor microenvironment (TME) cues (14). Thus, systematic dissection of METTL14’s regulatory networks across distinct tumor types is a foundational prerequisite for unraveling its functional duality and advancing its clinical translation.

**Table 1:** The expression trend of METTL14 in various tumors, core targets or pathways, and biological functions.

Tumor type	Expression	Core downstream target or pathway	Biological function	References
Hepatocellular carcinoma	down	USP48/SIRT6, circFUT8	proliferation, migration, metabolism, drug resistance	(6, 28– 32)
Colorectal cancer		β -catenin, SOX4, EBI3	stemness, EMT, immune escape	(7, 33– 35)
Gastric cancer		PTEN, ATF5, TAF10	proliferation, migration, invasion	(36– 39)
Clear cell renal cell carcinoma		PI3K/AKT, BPTF, ZFP14, TRAF1	proliferation, metastasis, EMT, metabolism, drug resistance	(13, 40– 43)
Endometrial cancer		GPX4	ferroptosis	(44)
Thyroid cancer		SOCS3, RAD21, OIP5-AS1	proliferation, EMT, apoptosis	(11, 45, 46)
Pancreatic cancer	up	PERP, LINC00941, CDA, c-Myc	proliferation, metastasis, EMT, drug resistance	(8, 15, 47– 50)
Nasopharyngeal carcinoma		ANKRD22, AOC1	proliferation, metastasis	(51, 52)
Oral squamous cell carcinoma		eIF4G1, MALAT1, CALD1	autophagy, proliferation, metastasis	(53– 55)
Leukemia/Marrow Dysplasia Syndrome		mdm2/p53, PD-L1, SETBP1/PI3K/AKT	proliferation, immune escape	(17, 56– 61)
Glioma/Neuroblastoma/Medulloblastoma		PD-L1, circRNA_103239/YWHAH, FOXD1	proliferation, invasion, EMT, immune escape	(62– 68)
Osteosarcoma		MN1	proliferation, migration, drug resistance	(12)
Retinoblastoma		LINC00340, CDKN2A	proliferation, apoptosis	(69, 70)
Non-small cell lung cancer	up or down	LINC02747, SLC3A2, miR-19a-5p	proliferation, migration, drug resistance, ferroptosis	(71– 75) ↓ (9, 76, 77) ↑
Bladder cancer		USP38, Notch1, lncDBET	migration, EMT, metabolism	(78, 79) ↓ (10, 80) ↑
Prostate cancer		THBS1, miR-129-5p	angiogenesis, drug resistance	(81, 82) ↓ (83, 84) ↑
Breast cancer		YAP1, USP22, E2F1, CALCOCO1, STK11	proliferation, metastasis, stemness, metabolism, drug resistance	(85) ↓ (86, 87) ↑
Cervical cancer		FTH1, AMPK, CYP1B1	proliferation, metastasis, ferroptosis, glycolysis, immune escape	(88) ↓ (89) ↑
Ovarian cancer		SMC4, TROAP	proliferation, migration, apoptosis	(90) ↓ (91– 93) ↑
Melanoma		SCD1, RUNX2, FAT4	migration, invasion, metabolism	(94) ↓ (95, 96) ↑

PERP, p53 effector related to PMP-22; CDA, cytidine deaminase; USP48, ubiquitin-specific protease 48; SIRT6, silent information regulator transcript 6; SOX4, SRY-box transcription factor 4; EBI3, epstein-barr virus induced 3; PTEN, phosphatase and tensin homolog; ATF5, activating transcription factor 5; TAF10, TATA-box binding protein associated factor 10; BPTF, bromodomain PHD finger transcription factor; ZFP14, zinc finger protein 14; TRAF1, TNF receptor associated factor 1; THBS1, thrombospondin 1; FTH1, ferritin heavy chain 1; CYP1B1, cytochrome P450 family 1 subfamily B member 1; SMC4, Structural maintenance of chromosomes 4; TROAP, trophinin associated protein; YAP1, yes-associated protein 1; E2F1, E2F transcription factor 1; CALCOCO1, calcium binding and coiled-coil domain 1; STK11, serine/threonine kinase 11; SLC3A2, solute carrier family 3 member 2; ANKRD22, ankyrin repeat domain 22; AOC1, amine oxidase copper containing 1; SOCS3, suppressor of cytokine signaling 3; RAD21, RAD21 cohesin complex component; OIP5-AS1, OIP5 antisense RNA 1; eIF4G1, eukaryotic translation initiation factor 4 gamma 1; MALAT1, metastasis associated lung adenocarcinoma transcript 1; CALD1, caldesmon 1; SETBP1, SET binding protein 1; MTSS1, MTSS I-BAR domain containing 1; YWHAH, Tyrosine 3-monooxygenase/tryptophan 5-monooxygenase activation protein eta; ASCL1, achaete-scute family bHLH transcription factor 1; FOXD1, forkhead box D1; SCD1, stearoyl-CoA desaturase 1; RUNX2, RUNX family transcription factor 2; FAT4, FAT atypical cadherin 4; MN1, meningioma 1; CDKN2A, cyclin dependent kinase inhibitor 2A.

### 3.1 Tumor-suppressive roles of METTL14

In the following tumor types, accumulating evidence supports a tumor-suppressive function of METTL14, where its downregulation or functional loss correlates with malignant tumor progression.

#### 3.1.1 Hepatocellular carcinoma

In hepatocellular carcinoma (HCC), METTL14 displays a distinct expression signature and participates in a highly orchestrated regulatory network. Accumulating evidence demonstrates that METTL14 is consistently downregulated in both HCC cell lines and primary tumor tissues. Functional studies show that forced METTL14 expression suppresses HCC cell proliferation and migration by targeting the hsa-miR-205/ZIK1 axis, supporting its role as a bona fide tumor suppressor (6). Moreover, METTL14 exerts broad anti-tumorigenic effects across multiple biological processes—including metabolic reprogramming and cell cycle control—through site-specific m^6^A methylation of functionally critical mRNAs.

With regard to metabolic regulation, Lutao Du et al. (28) reported that METTL14 installs m^6^A marks on ubiquitin-specific protease 48 (USP48) mRNA, thereby enhancing its transcript stability and promoting USP48 protein expression. This activates the METTL14–USP48–SIRT6 signaling axis, leading to suppression of glycolysis and inhibition of lipid metabolism reprogramming—two hallmarks of malignant transformation—ultimately impeding HCC initiation and progression. At the cell cycle level, recent work (29) revealed that loss of METTL14 stabilizes Fam32a mRNA, resulting in aberrant alternative splicing of Cdkn1a (p21) and accelerated G1/S transition, thereby fueling uncontrolled proliferation. Collectively, these findings underscore METTL14’s central role in preserving cellular homeostasis via precise post-transcriptional control.

METTL14 expression itself is subject to multilayered upstream regulation. Xin Tang et al. (30) identified that the solute carrier SLC27A5 and the RNA-binding protein PABPC1 cooperatively bind the 3′UTR of METTL14 mRNA to modulate its alternative splicing and polyadenylation, thereby increasing METTL14 abundance and suppressing the stemness properties of liver cancer stem cells. This delineates a key post-transcriptional mechanism through which METTL14 is positively regulated to exert tumor-suppressive functions.

Notably, METTL14’s functional output in HCC exhibits marked context dependency, particularly within the tumor microenvironment (TME). For instance, HCC cells internalize exosomal hsa-miR-628-5p secreted by M1-polarized macrophages, which directly targets and represses METTL14 expression (31). Mechanistically, nuclear METTL14 deposits m^6^A modifications on circFUT8, facilitating its YTHDC1-dependent nuclear export; once in the cytoplasm, circFUT8 acts as a competitive endogenous RNA (ceRNA) to sequester hsa-miR-552-3p, thereby derepressing CHMP4B expression and enhancing HCC cell invasion and stemness (31). While this exemplifies the canonical ceRNA function of circRNAs, emerging evidence indicates that numerous circRNAs are translatable and encode functional micropeptides or proteins with defined oncogenic or tumor-suppressive activities in proliferation, metastasis, and therapy resistance (98).

Conversely, METTL14 overexpression has also been linked to diminished sensitivity of HCC cells to regorafenib via activation of the CHOP-mediated unfolded protein response pathway (32), highlighting its potential dual functionality in therapeutic contexts. Taken together, these findings establish METTL14 as a pivotal, context-sensitive regulatory hub in HCC—subject to intricate transcriptional, post-transcriptional, and microenvironmental modulation—and position its m^6^A-dependent regulatory network not only over canonical mRNAs but also across diverse non-coding RNA species, including circRNAs, to orchestrate multi-tiered control of gene expression.

#### 3.1.2 Colorectal cancer

METTL14 serves as a central regulator in colorectal carcinoma (CRC), critically influencing tumorigenesis and progression, molecular subtyping, therapeutic response, and tumor microenvironment (TME) remodeling. Its downregulation and functional impairment are recurrent features across multiple stages of CRC malignant evolution.

Accumulating evidence demonstrates that METTL14 is consistently downregulated in CRC tissues compared with normal colonic epithelium. Mechanistically, METTL14 installs m^6^A modifications on β-catenin mRNA, impairing its nuclear translocation and thereby attenuating cancer stemness maintenance (7). At the transcriptional regulatory level, METTL14 targets the SOX4 transcript for m^6^A deposition, leading to reduced mRNA stability, diminished SOX4 protein expression, and consequent suppression of epithelial–mesenchymal transition (EMT) and downstream PI3K/Akt pathway activation (33).

Beyond m^6^A-dependent epitranscriptomic control, RNA stability is subject to direct modulation by RNA-binding proteins (RBPs). Notably, FXR1 functions as an oncogenic RBP in multiple malignancies: in esophageal squamous cell carcinoma, it binds to and accelerates the decay of tumor suppressor mRNAs—including PDZK1IP1 and ATOH8—thereby promoting tumor progression (99, 100); in lung and ovarian cancers, FXR1 acts as a context-dependent “molecular switch” that coordinately regulates the turnover of key oncogenic (e.g., c-MYC) and tumor-suppressive (e.g., p21) transcripts (101). These findings highlight that METTL14-mediated m^6^A modification and RBP-driven post-transcriptional regulation—exemplified by FXR1—do not operate in isolation but rather converge into an integrated, multilayered network governing CRC gene expression dynamics.

The functional impact of METTL14 exhibits marked subtype specificity. In CRC with wild-type p53 (WT-p53), the p53 protein directly interacts with the METTL14 promoter. This interaction enhances METTL14 transcriptional activity. This interaction establishes METTL14 as a crucial downstream mediator of p53’s tumor-suppressive functions, suggesting that METTL14 could be a potential target for precision therapies in this patient subgroup (97). In more aggressive KRASG12D-mutant CRC, the METTL14/LINC02159/FOXC2 signaling axis has been shown to modulate tumor cell sensitivity to the MRTX1133 inhibitor, providing a novel strategy to overcome resistance to targeted therapies (34). Furthermore, in colitis-associated colorectal cancer (CAC), vitamin D, via its receptor (VDR), directly binds to the METTL14 promoter to upregulate its expression. This leads to m^6^A modification of key targets such as SOX4 and Drosha, exerting chemopreventive effects and revealing, for the first time, the protective role of the VDR–METTL14 axis in CAC pathogenesis (102).

METTL14 shows dual and context-specific roles in the tumor immune microenvironment. In the case of rectal cancer, elevated levels of METTL14 are significantly associated with heightened infiltration of immune cells and a better prognosis (35). Conversely, silencing METTL14 in tumor-associated macrophages (TAMs) within colorectal cancer (CRC) reduces the degradation of EBI3 mRNA mediated by m^6^A modification. This leads to accumulation of EBI3, which inhibits anti-tumor T-cell responses and promotes immune evasion. These findings highlight METTL14’s immunosuppressive role in specific immune contexts (14). From a therapeutic standpoint, low METTL14 expression diminishes m^6^A modification of pri-miR-17, leading to aberrant upregulation of miR-17-5p, inhibition of the mitochondrial fusion protein MFN2, and consequent resistance to 5-fluorouracil chemotherapy (103).

Beyond these roles, METTL14 is deeply involved in determining cancer cell fate. The natural compound curcuminone induces ferroptosis in CRC cells by upregulating the METTL14/YTHDF2 axis, promoting m^6^A modification and degradation of ferroptosis-related transcripts (104). Additionally, METTL14 exerts significant control over glycolytic metabolism, thereby influencing tumor metabolic reprogramming (105). Environmental risk factors such as smoking also exploit this pathway: the tobacco-specific nitrosamine NNK hijacks the METTL14/YTHDF2 axis to stabilize TMUB1 mRNA, leading to aberrant activation of the AKT signaling pathway and driving CRC development and progression (106).

#### 3.1.3 Gastric cancer

METTL14 plays a predominant tumor suppressor role in gastric cancer, where its downregulation is strongly associated with tumor progression and poor prognosis. It exerts multi-layered regulatory effects by targeting multiple critical molecules. The study by Qi Yao et al. (36) first demonstrated that in stomach adenocarcinoma (STAD), METTL14 enhances the stability of PTEN mRNA through m^6^A modification, thereby suppressing tumor growth and metastasis. Given the pivotal role of PTEN in modulating chemotherapy resistance and immune responses (107), whether METTL14 influences STAD treatment sensitivity via PTEN regulation remains an important avenue for future investigation.

In gastric cancer (GC), METTL14 mediates m^6^A modification of TAF10 mRNA to promote its degradation, leading to inhibition of tumor cell proliferation, migration, and invasion (37). Furthermore, METTL14 regulates the miR-30c-2-3p/AKT1S1 signaling axis by depositing m^6^A marks on circORC5, ultimately attenuating GC cell growth and invasive capacity (38). In contrast, work by Peiling Zhang et al. revealed that loss of METTL14 enhances m^6^A modification of ATF5 mRNA, stabilizing its expression and activating the WDR74/β-catenin signaling pathway, which confers stem cell-like properties to cancer cells and drives malignant progression (39). Collectively, these findings establish that METTL14 forms a multi-node tumor suppressive network in gastric cancer—orchestrated through key targets including PTEN, ATF5, TAF10, and circORC5—providing both a mechanistic foundation and novel therapeutic targets for precision oncology in GC.

#### 3.1.4 Renal cell carcinoma

METTL14 plays a pivotal regulatory role in the initiation, progression, metastasis, and therapeutic resistance of renal cell carcinoma, particularly clear cell renal cell carcinoma (ccRCC), with its dysregulated expression and multi-layered molecular mechanisms increasingly being elucidated.

In ccRCC, METTL14 exhibits specific downregulation and is significantly correlated with tumor malignancy and advanced disease stages. Accumulating evidence indicates that upregulation of METTL14 suppresses PI3K/AKT signaling pathway activity, thereby markedly impairing the *in vitro* proliferative and migratory capacities of ccRCC cells, supporting its role as a tumor suppressor (40, 41). At the molecular level, Zhang et al. (13) demonstrated that METTL14 mediates m^6^A modification of BPTF mRNA to enhance its stability, leading to activation of glycolytic metabolic reprogramming and providing energetic support for tumor dissemination. Concurrently, METTL14 inhibits EMT and metastatic potential through its downstream effector ZFP14 (42) and via m^6^A-dependent post-transcriptional regulation of ITGB4 (43), underscoring its functional significance as a metastasis-suppressing factor in ccRCC. Nevertheless, the precise roles of METTL14 in modulating the tumor immune microenvironment, maintaining cancer stem cell stemness, and regulating apoptotic signaling pathways remain incompletely understood and warrant further investigation through integrated multi-omics approaches and validation in well-characterized clinical cohorts.

From a therapeutic perspective, sunitinib remains a first-line targeted agent for ccRCC; however, the development of drug resistance poses a major clinical challenge. Chen et al. (16) revealed that METTL14 enhances TRAF1 mRNA stability through m^6^A modification, resulting in sustained activation of anti-apoptotic and angiogenesis-promoting signaling pathways, ultimately contributing to reduced sensitivity to sunitinib. Collectively, these findings comprehensively delineate the multi-dimensional tumor-suppressive functions of METTL14 in ccRCC, not only advancing our mechanistic understanding of its biological roles but also offering novel therapeutic targets and strategic insights for overcoming resistance to targeted therapies.

#### 3.1.5 Endometrial cancer

The regulatory mechanisms and biological functions of METTL14 in endometrial cancer (EC) remain poorly understood and are still at an early stage of investigation. Current evidence indicates that protein arginine methyltransferase 3 (PRMT3) interacts with METTL14 and catalyzes its arginine methylation modification; inhibition of PRMT3 significantly upregulates METTL14 expression. Subsequently, elevated METTL14 promotes the YTHDF2-dependent m^6^A-mediated degradation of glutathione peroxidase 4 (GPX4) mRNA, resulting in the accumulation of mitochondrial lipid peroxidation and ultimately inducing ferroptosis in EC cells (44). Nevertheless, it remains unclear whether METTL14 contributes to EC progression through additional downstream targets or signaling pathways, how its expression correlates with clinical pathological characteristics, and what therapeutic potential it may hold—these aspects warrant further systematic investigation.

#### 3.1.6 Thyroid carcinoma

METTL14 exerts prominent tumor-suppressive functions in thyroid cancer (TC), yet its mechanism of action is characterized by multi-targeted and multi-pathway regulation. In recent years, multiple studies have delineated its regulatory network from distinct angles. Regarding the regulation of protein-coding genes, Ming Zhou et al. (45) demonstrated that METTL14 upregulates the expression of the tumor suppressor SOCS3 via m6A modification, thereby inhibiting the activation of the JAK2/STAT3 signaling pathway—ultimately suppressing thyroid cancer cell proliferation, EMT, and promoting apoptosis. Similarly, Yinzhe Ge et al. (46) revealed that METTL14 mediates m6A modification of RAD21 mRNA to reduce its stability, downregulating RAD21 expression and thus impeding the malignant progression of TC. Xiaoping Zhang et al. (11) found that METTL14 can directly bind to the lncRNA OIP5-AS1 and negatively regulate its expression through m6A modification. As a molecular sponge for miR-98, OIP5-AS1 sequesters miR-98, relieving its inhibitory effect on the target gene ADAMTS8 and subsequently activating the EGFR/MEK/ERK signaling pathway, which ultimately promotes the proliferation, migration, and invasion of papillary thyroid cancer (PTC) cells.

In summary, accumulated research highlights METTL14’s pivotal role as a tumor suppressor in TC. It modulates various downstream targets, such as SOCS3, RAD21, and OIP5-AS1, through m6A modification-dependent mechanisms. Furthermore, it influences critical signaling pathways, such as JAK2/STAT3 and EGFR/MEK/ERK, and thereby impedes the progression of TC. These insights not only enhance our understanding of m6A RNA modification’s role in TC pathogenesis but also provide a theoretical basis for developing METTL14-targeted therapies, such as METTL14 agonists. This approach holds considerable clinical relevance, especially for patients with advanced or drug-resistant forms of TC. Future studies must validate these regulatory networks in larger clinical cohorts. Additionally, their potential for translation into clinical practice should be assessed.

### 3.2 Oncogenic regulatory roles of METTL14

In the following tumor types, accumulating evidence supports an oncogenic function of METTL14, where its overexpression correlates with malignant tumor progression and unfavorable clinical prognosis.

#### 3.2.1 Pancreatic cancer

METTL14, an essential component of the m^6^A methyltransferase complex, plays a key role in regulating the onset, progression, and treatment resistance of pancreatic cancer (PC). Consequently, it has become a prominent focus in current oncological research. A growing body of evidence suggests that METTL14 is markedly overexpressed in pancreatic cancer tissues, and its expression is strongly associated with advanced AJCC staging, poor tumor differentiation, and lymphatic metastasis. It has been established as an independent prognostic marker indicative of unfavorable clinical outcomes (8, 47), with its aberrant overexpression being closely associated with more aggressive tumor characteristics.

At the molecular level, METTL14 drives pancreatic cancer progression through multifaceted mechanisms mediated by m^6^A modification of diverse RNA substrates. Specifically, METTL14 recognizes the 3’UTR of PERP mRNA and facilitates its degradation via the m^6^A reader YTHDF2, thereby attenuating PERP-mediated tumor suppression and promoting tumor cell proliferation and metastasis (47). In addition, METTL14 plays a crucial role in the stabilization of the long non-coding RNA LINC00941, which depends on IGF2BP2.This interaction triggers the activation of EMT, thereby increasing the invasive potential of cancer cells (48). Additionally, the CLK1–SRSF5 signaling axis regulates alternative splicing of METTL14 pre-mRNA, particularly by suppressing the generation of exon 10-skipped isoforms, thus preserving its oncogenic function (49). Furthermore, METTL14 promotes tumorigenesis by activating critical oncogenic pathways such as c-Myc, modulating downstream gene expression to accelerate cell cycle progression and suppress apoptosis, thereby fueling malignant progression (50).

The persistence of resistance to first-line chemotherapeutic agents such as gemcitabine significantly hampers the prognosis of pancreatic cancer. Importantly, Congjun Zhang and colleagues (15) conducted research revealing that METTL14 is significantly overexpressed in pancreatic cancer cells. This overexpression is associated with resistance to gemcitabine. This upregulation depends on p65 (NF-κB), which directly interacts with and activates the METTL14 promoter. This upregulation enhances the expression of cytidine deaminase (CDA), which accelerates the metabolic inactivation of gemcitabine via deamination, ultimately diminishing drug efficacy. Collectively, these findings establish METTL14 as a central regulatory node in pancreatic cancer, orchestrating a novel m^6^A-dependent gemcitabine resistance pathway by modulating key metabolic enzymes such as CDA. These insights provide a robust theoretical foundation for developing therapeutic strategies aimed at reversing chemoresistance by targeting METTL14 or its downstream effectors.

#### 3.2.2 Nasopharyngeal carcinoma

In tissues affected by nasopharyngeal carcinoma (NPC), METTL14 expression increases notably. Moreover, higher METTL14 levels significantly correlate with lower overall survival, increased lymph node metastasis, and advanced clinical stages. These clinical features are important indicators of a poor prognosis (51, 52). Accumulating evidence indicates that METTL14 functions as a critical oncogenic driver by enhancing the proliferative and invasive capabilities of NPC cells, thereby serving as a central regulator of tumor malignancy.

Mechanistically, METTL14 promotes NPC progression primarily through m^6^A methylation-mediated regulation of two key signaling axes. First, METTL14 upregulates the expression of ankyrin repeat domain 22 (ANKRD22) via m^6^A-dependent post-transcriptional control. Elevated ANKRD22 increases intracellular acetyl-CoA levels, which serve as a substrate donor for histone acetylation, leading to H3K27ac modification. This epigenetic mark enhances the transcriptional activity of tumor-promoting genes and simultaneously transactivates the METTL14 promoter, establishing a self-reinforcing METTL14/ANKRD22/H3K27ac positive feedback loop that sustains the malignant phenotype of NPC cells (51). Second, as a core component of the m^6^A methyltransferase complex, METTL14 mediates m^6^A modification of amine oxidase copper-containing 1 (AOC1) mRNA, thereby enhancing its stability, preventing degradation, and ultimately increasing AOC1 protein expression. This regulatory axis cooperatively drives NPC cell proliferation and metastasis (52).

Collectively, METTL14 exerts a pivotal role in NPC progression through dual mechanisms: a lipid metabolism–histone modification positive feedback loop and the stabilization of AOC1 mRNA. Its overexpression represents a promising biomarker for predicting adverse outcomes in NPC patients. Future studies should aim to precisely map the m^6^A modification sites on ANKRD22 and AOC1 mRNA regulated by METTL14, elucidate the detailed molecular mechanisms underlying the METTL14/ANKRD22/H3K27ac regulatory circuit, and explore the therapeutic potential of targeting METTL14 with small-molecule inhibitors or m^6^A-modulating agents in precision medicine strategies for NPC.

#### 3.2.3 Oral squamous cell carcinoma

Recent studies have demonstrated that the methyltransferase METTL14 exerts multifaceted oncogenic functions in the initiation and progression of oral squamous cell carcinoma (OSCC) through mediating m6A modifications of diverse downstream targets. Fang Wang et al. (53) reported that METTL14 enhances the m6A modification of eukaryotic translation initiation factor 4G1 (eIF4G1), thereby promoting its expression and activating autophagy, which together establish a “METTL14–m6A–eIF4G1–autophagy” regulatory axis that drives the malignant progression of OSCC. Jinli Li et al. (54) further demonstrated that METTL14 mediates m6A modification of the long non-coding RNA MALAT1, increasing its transcript stability; upregulated MALAT1 subsequently acts as a competitive endogenous RNA (ceRNA) to sequester miR-224-5p, thereby alleviating its repression of the histone demethylase KDM2A and ultimately activating proliferative signaling pathways. In addition, Ruixue Chen et al. (55) showed that METTL14 upregulates calmodulin-like protein 1 (CALD1) expression in an m6A-dependent manner, directly enhancing the proliferative and metastatic capacities of OSCC cells. Collectively, these findings underscore the central role of METTL14 as a key epigenetic regulator that modulates OSCC pathogenesis through coordinated regulation of both coding and ncRNAs. Nevertheless, the precise m6A modification sites within these pathways, the identity and function of reader proteins involved in recognizing these marks, and the comprehensive architecture of downstream signaling networks remain poorly defined. Furthermore, as current evidence is predominantly derived from preclinical models, the clinical translatability of these findings necessitates validation through large-scale cohort studies and rigorous functional investigations.

#### 3.2.4 Leukemia/marrow dysplasia syndrome

Peihua Zhang and colleagues (56) found that elevated METTL14 expression significantly correlates with poor prognosis in juvenile myelomonocytic leukemia (JMML).Using the KrasG12D/+ mutant mouse model, the study showed that genetic deletion of METTL14 reduces the expression of autophagy-related genes Atg5 and Atg9a in an m^6^A-dependent manner. Reduced METTL14 activity lowers autophagy levels in hematopoietic stem and progenitor cells (HSPCs), thereby inhibiting leukemia progression. Notably, this study provides pioneering evidence that combining m^6^A and MEK inhibitors synergistically inhibits JMML cell proliferation. This finding suggests an innovative dual-pathway therapeutic strategy for Kras-driven malignancies.

In acute myeloid leukemia (AML), accumulating evidence has elucidated the multi-layered regulatory mechanisms governed by METTL14. Lina Sang et al. (57) established that the METTL3/METTL14 complex enhances mdm2 mRNA stability through m^6^A modification, leading to suppression of the p53 signaling pathway and defining a “METTL3/METTL14–m^6^A–mdm2–p53” regulatory axis. Mengmeng Zhang et al. (58) reported that METTL14-mediated m^6^A methylation stabilizes TCP1 transcripts, promoting AML cell proliferation and tumorigenesis, potentially via activation of the PI3K/AKT signaling pathway. Meng Wang et al. (17) revealed, for the first time, that METTL14 modulates macrophage polarization through the “METTL14–PD-L1” signaling axis, driving the tumor microenvironment toward an immunosuppressive M2 phenotype—a pro-tumorigenic effect that can be reversed by anti-PD-L1 therapy. Yulun Zhong et al. (59) identified the PRMT5–METTL14 regulatory axis as a molecular link between protein arginine methylation and RNA m^6^A methylation, highlighting a functional crosstalk that opens new avenues for combinatorial targeting of epitranscriptomic modifications in cancer therapy.

In myelodysplastic syndrome (MDS), Lingxu Jiang et al. (60) demonstrated that METTL14 expression is significantly upregulated and positively correlated with disease risk stratification. Mechanistically, METTL14 forms a complex with METTL3 to enhance m^6^A modification of SETBP1 mRNA, thereby increasing transcript stability and activating the PI3K-AKT signaling pathway, which promotes aberrant proliferation of MDS cells. In an environmentally induced model of hematological malignancies, Chao Wu et al. (61) reported that prolonged benzene exposure induces METTL14 overexpression in mouse LSK cells (Lin^-^Sca-1^+^c-Kit^+^), leading to elevated m^6^A levels on key genes such as mTOR and GFI1, activation of the mTOR-AKT signaling pathway, and consequent malignant proliferation of bone marrow cells.

Collectively, METTL14 plays an integral role in the onset and progression of hematological cancers. It functions through various molecular mechanisms, including the regulation of autophagy, key signaling pathways, and the tumor microenvironment. Strategic targeting of METTL14 and its associated epitranscriptomic regulatory network offers a promising therapeutic avenue, enabling the development of novel strategies for managing hematological malignancies.

#### 3.2.5 Glioma/neuroblastoma/medulloblastoma

Research has indicated that METTL14, a core component of the m6A methyltransferase complex, is involved in key regulatory functions related to glioma formation, tumor progression, and treatment responses. Moreover, its expression is tightly regulated by upstream epigenetic factors. For instance, the histone methyltransferase SETD2 catalyzes H3K36me3 deposition to positively regulate METTL14 transcription, thereby establishing a “histone–RNA modification” cascade regulatory axis (63). METTL14 exhibits context-dependent functional plasticity: during oncolytic virus therapy, it enhances antiviral immunity by stabilizing ISG15 mRNA, yet it can also be degraded by viral proteins to facilitate immune evasion—highlighting its potential as a key target for improving virotherapy efficacy (64). Within the tumor immune microenvironment, METTL14 stabilizes PD-L1 mRNA via m6A modification, directly promoting glioblastoma proliferation, invasion, and immune escape (65). At the downstream mechanistic level, METTL14 mediates m6A modification of the circular RNA circRNA_103239, leading to activation of the miR-182-5p/MTSS1 signaling pathway and subsequent suppression of EMT, thereby exerting tumor-suppressive effects (66).

Collectively, these findings underscore the central role of METTL14 in the epitranscriptomic regulatory network of glioma, revealing a multilayered, coordinated interplay among “histone modification–RNA modification–non-coding RNA” and providing a robust theoretical foundation for developing novel therapeutic strategies targeting m6A-dependent pathways. Nevertheless, the microenvironment-specific functions of METTL14, its complete regulatory circuitry, and the translational bridge to clinical applications remain to be fully elucidated.

The study by Jianwei Wang et al. (62) demonstrated that in high-risk neuroblastoma, METTL14 expression is positively regulated by the transcription factors ETS1 and YY1 and promotes mRNA degradation of the downstream target gene YWHAH via an m6A-YTHDF1-dependent mechanism, thereby activating the PI3K/AKT signaling pathway and driving tumor proliferation and invasion. Complementarily, Ting Hu et al. (67) identified an additional METTL14-m6A-dependent oncogenic pathway in neuroblastoma: m6A modification functions as a “molecular switch” that facilitates the binding of RNA-binding proteins HNRNPA2B1 and HNRNPR to the 5’ and 3’ untranslated regions (UTRs) of ASCL1 mRNA, respectively, and cooperates with IGF2BP1 to enhance the mRNA stability of this transcription factor, thereby exacerbating tumor malignancy and establishing a defined “METTL14–HNRNPA2B1/HNRNPR–ASCL1” regulatory axis. Yantao Liu and colleagues (68) studied the tumor microenvironment in sonic hedgehog subtype medulloblastoma (SHH-MB). They demonstrated that exosomes from tumor-associated macrophages (TAMs) contribute to downregulating METTL14.This downregulation reduces global m6A methylation levels, which in turn relieves the transcriptional repression on the transcription factor FOXD1.This upregulation of FOXD1 suppresses chemokine secretion, inhibits CD8^+^ T cell infiltration, and fosters an immunosuppressive microenvironment; notably, targeting FOXD1 significantly enhances the therapeutic response to PD-1 inhibitors.

Collectively, these three studies systematically elucidate how METTL14-mediated m6A modification drives tumor progression in pediatric nervous system tumors through both cell-autonomous mechanisms—such as activation of the YWHAH/PI3K-AKT axis and stabilization of ASCL1—and non-cell-autonomous mechanisms—including response to TAM-derived signals and FOXD1-mediated remodeling of the immune microenvironment. These findings not only deepen our understanding of the complexity within epitranscriptomic regulatory networks but also provide a robust theoretical foundation and novel potential therapeutic targets for developing m6A-targeted interventions and their rational combination with immunotherapy.

#### 3.2.6 Osteosarcoma

Hong-Bo Li and colleagues (12) conducted an investigation that thoroughly elucidated the significant function and molecular mechanisms of METTL14-mediated m6A modification in the malignant progression of osteosarcoma (OS). It was demonstrated that METTL14 is upregulated in OS and regulates the expression of the proto-oncogene MN1 through an m6A-dependent mechanism: METTL14 deposits m6A modifications within the coding sequence (CDS) of MN1 mRNA, which are specifically recognized by the RNA-binding protein IGF2BP2, thereby enhancing MN1 mRNA stability and increasing its translational efficiency, ultimately promoting tumor progression and conferring resistance to all-trans retinoic acid (ATRA) chemotherapy. However, the cell type-specific distribution of this regulatory axis within the OS tumor microenvironment, as well as its potential for synergistic interaction with current therapeutic regimens, remains to be fully elucidated. Although the METTL14–IGF2BP2–MN1 axis holds significant promise for clinical translation, its predictive power as a prognostic biomarker requires validation in prospective, large-scale cohort studies, and targeted therapeutic strategies against this pathway remain to be developed. These findings not only provide novel insights into the molecular pathogenesis of osteosarcoma but also establish a foundation for developing potential therapeutic targets and biomarkers aimed at overcoming ATRA resistance and improving patient outcomes.

#### 3.2.7 Retinoblastoma

Although the regulatory role of METTL14-mediated m6A modification has been well established in various cancers, its molecular mechanisms in retinoblastoma (RB) remain incompletely understood. Recent studies by Jing Chen et al. (69, 70) demonstrate that METTL14 drives tumor progression in RB through multiple m6A-dependent pathways. On one hand, METTL14 deposits m6A modifications on the transcript of the long non-coding RNA LINC00340, enhancing its RNA stability, thereby activating the Notch signaling pathway and promoting cell proliferation while suppressing apoptosis (69). On the other hand, METTL14 upregulates CDKN2A expression via m6A modification, leading to inhibition of the p53 signaling pathway and exerting a pro-oncogenic effect (70). Together, these findings reveal a dual oncogenic mechanism of METTL14 in RB, highlighting its ability to promote tumor malignancy through modulation of distinct downstream targets—LINC00340 and CDKN2A—and key signaling axes—Notch and p53. These results not only provide novel insights into the epitranscriptomic regulatory network in retinoblastoma but also suggest that targeting METTL14 or its downstream effectors may represent a promising therapeutic strategy. Nevertheless, potential crosstalk between these pathways and the clinical translatability of this regulatory axis warrant further investigation.

### 3.3 Tumor types with controversial or context-dependent functions of METTL14

In the following tumor types, conflicting findings have been reported regarding the biological role of METTL14. Alternatively, its function within a single tumor entity is highly context-dependent, being tightly regulated by factors such as molecular subtypes, TME components, and cellular states.

#### 3.3.1 Lung cancer

Research on METTL14 in lung cancer has mainly focused on non-small cell lung cancer (NSCLC). This subtype accounts for over 85% of lung cancer cases, but the expression and regulatory functions of METTL14 remain controversial. These discrepancies may result from various factors, such as different pathological subtypes (e.g., lung adenocarcinoma and lung squamous cell carcinoma), tumor stage, genetic variations, and detection methods. Increasing evidence shows that METTL14 often decreases in NSCLC, and this reduction significantly correlates with poor patient outcomes, highlighting its critical role in tumor aggressiveness (71–73). Mechanistically, downregulation of METTL14 reduces m^6^A modification of LINC02747. This reduction increases LINC02747 RNA stability, which promotes tumor cell proliferation and migration by activating the CDK4/CyclinD1 complex and the PI3K/Akt signaling pathway (71).Additionally, METTL14 regulates ferroptosis by modulating the m^6^A-dependent stability of SLC3A2 mRNA (74). In EGFR-mutant NSCLC, reducing METTL14 expression leads to a widespread decrease in m^6^A levels and contributes to resistance to osimertinib (75).

Conversely, several studies have reported upregulation of METTL14 in NSCLC, with high expression linked to increased tumor invasiveness and therapy resistance (9, 76, 77). For example, elevated METTL14 enhances m^6^A modification of the miR-19a-5p/RBM24/AXIN1 regulatory axis, thereby increasing resistance to cisplatin in NSCLC cells (9). This paradoxical phenomenon—where both upregulation and downregulation of METTL14 promote tumorigenesis—may stem from its context-dependent regulation of downstream targets: under low-expression conditions, METTL14 indirectly facilitates cancer progression by stabilizing tumor suppressor transcripts that escape m^6^A-mediated decay; whereas under high-expression conditions, it directly drives oncogenesis by enhancing the m^6^A-dependent stability and translation of oncogenic mRNAs.

Collectively, METTL14 plays a multifaceted role in NSCLC by regulating critical target genes such as LINC02747 and SLC3A2, as well as key signaling pathways including PI3K/Akt and miR-19a-5p/RBM24/AXIN1, thereby influencing tumor proliferation, metabolic reprogramming, and therapeutic resistance. Future investigations should integrate single-cell sequencing and spatial transcriptomics with other cutting-edge technologies to systematically dissect the dynamic and subtype-specific regulatory networks of METTL14 across disease stages, laying the foundation for its clinical translation as a precision therapeutic target in NSCLC.

#### 3.3.2 Bladder cancer

In bladder cancer (BCa), METTL14 exhibits a functionally controversial role: while accumulating evidence supports its tumor-suppressive activity, emerging studies also indicate that it may act as an oncogene in specific contexts. This duality likely arises from tumor heterogeneity and the complexity of downstream regulatory networks.

Multiple studies have demonstrated that METTL14 is downregulated in certain BCa tissues and tumor-initiating cells (TICs), with its expression level inversely correlated with tumor invasion and lymph node metastasis, underscoring its potential tumor suppressor function (78, 79). Mechanistically, METTL14 enhances the stability of USP38 mRNA through an m^6^A-YTHDF2-dependent pathway, thereby EMT and impairing cell migration capacity (78). Furthermore, the METTL14/m^6^A axis cooperatively inhibits the Notch1 signaling pathway, blocking malignant transformation of bladder epithelium and compromising the maintenance of TIC stemness, thus providing a mechanistic rationale for targeting cancer stem cells (79). Additionally, non-coding RNAs such as miR-3165 can negatively regulate METTL14 expression by binding to its 3’UTR, leading to attenuation of its tumor-suppressive effects—highlighting this regulatory axis as a potential therapeutic target (78).

Conversely, other reports show that METTL14 is upregulated in highly aggressive BCa cell lines (e.g., UMUC3) and subsets of clinical specimens, where its high expression correlates with advanced tumor grade and poor prognosis (10, 80). In these contexts, METTL14 mediates m^6^A modification of lncDBET, promoting FABP5-driven lipid metabolic reprogramming, which fuels tumor proliferation and invasion, thereby establishing a pro-tumorigenic METTL14/lncDBET/FABP5 signaling axis (10).

The conflicting findings regarding METTL14’s role in BCa may be attributed to several factors, including tumor heterogeneity, context-dependent downstream effectors and signaling pathways, and inherent biases across different experimental models. To resolve these discrepancies, future research should integrate large-scale clinical cohorts with single-cell transcriptomic and epigenomic profiling to precisely delineate the functional landscape of METTL14. Such efforts are essential for harnessing its potential as a biomarker or therapeutic target in precision oncology for bladder cancer.

#### 3.3.3 Prostate cancer

The biological function of METTL14 in prostate cancer is characterized by a controversial dual or multi-dimensional regulatory role. On one hand, multiple studies have uncovered its tumor-promoting effects. For example, Yongjie Wang et al. (83) demonstrated that METTL14 degrades the mRNA of the tumor suppressor gene THBS1 via an m^6^A-YTHDF2-dependent mechanism, thereby facilitating angiogenesis and tumor proliferation, and establishing the “METTL14-YTHDF2-THBS1” oncogenic regulatory axis. In the context of chemotherapy resistance, Cheng Wu et al. (84) further revealed the critical oncogenic role of METTL14, showing that it drives docetaxel resistance through the “E2F1-METTL14-miR-129-5p” axis and links DNA methylation with m^6^A modification in a cascade regulatory manner.

On the other hand, other studies suggest that METTL14 may exert tumor-suppressive effects or improve therapeutic outcomes in certain contexts. Zhuo Chen et al. (81) innovatively connected the anesthetic propofol with m^6^A regulation, finding that propofol inhibits prostate cancer progression by upregulating the long non-coding RNA TRHDE-AS1, and this process may involve METTL14-mediated m^6^A modification, implying a potential tumor-suppressive association of METTL14 in this pathway. Additionally, regarding treatment sensitivity, Qiwei Liu et al. (82) showed that upregulation of METTL14 expression enhances the sensitivity of prostate cancer cells to PARP inhibitors, providing a theoretical basis for METTL14 as a sensitization target in combination therapy.

In summary, existing studies collectively establish the core position of METTL14 in the regulatory network of prostate cancer, but its specific functional direction (oncogenic or tumor-suppressive) is highly dependent on the specific molecular context, regulatory axis, and therapeutic scenario. This contradiction underscores the complexity of its function. To clarify the underlying mechanisms and explore the feasibility of clinical translation, more in-depth functional validation and systematic preclinical studies are required to dissect each regulatory axis.

#### 3.3.4 Breast cancer

METTL14 exhibits a complex expression pattern and functional duality in breast cancer (BC), with its biological role demonstrating marked dependence on molecular subtypes and playing a critical role in tumor progression and therapeutic resistance. At the expression level, METTL14 levels vary significantly across different BC subtypes. In triple-negative breast cancer (TNBC), METTL14 is notably downregulated. This downregulation reduces the YTHDF2-mediated degradation of YAP1 mRNA. This reduction in degradation leads to an increase in YAP1 protein levels, which promotes cancer cell stemness and contributes to their chemotherapy resistance (85). Conversely, in estrogen receptor alpha (ERα)-positive breast cancer, METTL14 is upregulated and positively correlates with the expression of USP22 and ERα protein.METTL14 enhances the m^6^A modification and stability of USP22 and ERα mRNAs, thereby synergistically promoting the proliferation and migratory capacity of ERα-positive tumor cells (86). Notably, m^6^A methylation has been established as a key regulatory mechanism governing ubiquitination events in various cancers (108). Building on this insight, Bo Huang et al. (109) further demonstrated that under hypoxic conditions, tumor cell-derived and exosomal METTL14 promotes TNBC proliferation, metastasis, and glycolytic reprogramming by modulating TRIM16-mediated FGF7 ubiquitination and stabilizing FGF7 protein (109).

Beyond its role in driving tumor aggressiveness, METTL14 is also a pivotal regulator of acquired drug resistance in breast cancer. Its overexpression upregulates E2F1 through IGF2BP2-dependent m^6^A modification, activates cell cycle signaling pathways, and confers resistance to CDK4/6 inhibitors; notably, the novel peptide inhibitor WKYMVM can specifically target this axis and restore drug sensitivity (110, 111). Additionally, the METTL3/METTL14 complex regulates endoplasmic reticulum autophagy by mediating m^6^A modification of CALCOCO1 mRNA, and its inhibition significantly enhances the chemotherapeutic efficacy of paclitaxel (112). In HER2-positive breast cancer, the transcription factor RAD21 directly induces METTL14 expression, which in turn increases the m^6^A-dependent stability of STK11 mRNA, thereby promoting resistance to trastuzumab (87).

Collectively, METTL14 serves as a central regulatory node in breast cancer by orchestrating the expression of key genes—including YAP1, USP22, E2F1, CALCOCO1, and STK11—thereby influencing cancer stemness, metabolic reprogramming, and multidrug resistance. Future studies should leverage single-cell sequencing and spatial omics technologies to systematically dissect its context-specific regulatory networks across subtypes, accelerating its development as a predictive biomarker for therapy resistance and a promising therapeutic target.

#### 3.3.5 Cervical cancer

The expression pattern and clinical significance of METTL14 in cervical cancer (CC) remain controversial. Mateja Condic et al. (89) based on immunohistochemical analysis of 118 samples showed that high expression of METTL14 in the nucleus was significantly associated with shortened overall survival of patients; while Lijie Li et al. (88) found that the expression level of METTL14 and the overall m^6^A modification degree were significantly down-regulated in cervical cancer tissues, and its low expression was closely related to poor prognosis. The above differences may be due to different tumor stages, differences in detection methods, or the influence of the dynamic regulation characteristics of METTL14 during disease progression, which urgently need to be further verified through large-sample, multi-center studies.

At the molecular level, METTL14 contributes to CC progression through diverse mechanisms. Under sorafenib treatment, METTL14 promotes FTH1 mRNA degradation in an m^6^A-dependent manner, reducing its stability. This process enhances ferroptosis and inhibits PI3K/Akt signaling pathway activity, ultimately suppressing tumor proliferation and metastasis (88). Bingyu Wang et al. (113) showed that METTL14 activates the AMPK-glycolysis pathway, leading to lactic acid accumulation in the tumor microenvironment (TME). This accumulation induces M2 macrophage polarization and impairs antigen presentation, thereby facilitating immune evasion. Additionally, Qi Xie et al. (114) found that piRNA-14633 upregulates METTL14 expression and activity, which in turn targets CYP1B1 to enhance the proliferation, invasion, and migration of CC cells. These findings suggest that the piRNA-14633/METTL14/CYP1B1 signaling axis may represent a potential target for precision therapy.

In summary, although the expression characteristics of METTL14 in cervical cancer are not entirely consistent, its crucial role in regulating ferroptosis, metabolic reprogramming, and the tumor immune microenvironment has been confirmed by multiple studies, highlighting its significant potential as a therapeutic target, especially in combination with strategies targeting ferroptosis or reshaping the immune microenvironment.

#### 3.3.6 Ovarian cancer

The biological function of METTL14 in ovarian cancer (OC) remains controversial. Some studies support its oncogenic role, whereas others suggest it may act as a tumor suppressor. Evidence supporting the oncogenic role demonstrates that high METTL14 expression is significantly associated with reduced overall survival and promotes OC progression by enhancing cell proliferation and migration while suppressing apoptosis (91, 92). At the molecular level, the transcription factor FoxO1 activates METTL14 transcription, and in turn, METTL14 enhances the stability of SMC4 mRNA through m^6^A modification, thereby establishing a FoxO1/METTL14/SMC4 positive feedback regulatory loop that cooperatively drives tumor malignancy (93). In contrast, Yize Li et al. (90) reported a tumor-suppressive role for METTL14, showing that its low expression correlates significantly with shorter overall survival, and that METTL14 overexpression markedly inhibits the *in vitro* proliferative capacity of ovarian cancer cells. This effect is mediated through an m^6^A-dependent downregulation of TROAP, leading to suppression of cyclin D1, Survivin, and p-AKT signaling pathway activities. The observed functional variations may stem from distinct molecular subtypes of ovarian cancer, different disease stages, or variability in experimental models. Therefore, subsequent investigations ought to employ high-throughput sequencing methodologies to systematically delineate the downstream target network of METTL14.This approach will help to better understand the complex regulatory mechanisms of METTL14 in ovarian cancer.

#### 3.3.7 Melanoma

METTL14 exhibits a dual and context-dependent regulatory function in both the onset and progression of melanoma, and its effects vary significantly across tumor subtypes, target genes, and regulatory networks. In choroidal melanoma (CM), METTL14 mediates lipid metabolic reprogramming via the MAFG-METTL14-SCD1 axis, increasing cell membrane fluidity and thereby promoting tumor metastasis (95). On the other hand, METTL14 enhances RUNX2 expression via m6A methylation, stimulating the Wnt/β-catenin signaling pathway and promoting cellular migration and invasion (96). In contrast, in ocular melanoma, histone deacetylase inhibitors restore METTL14 expression and stabilize the mRNA of the tumor suppressor FAT4 in an m6A-YTHDF1-dependent manner, leading to tumor-suppressive effects (94). Collectively, these findings highlight the central regulatory role of METTL14 in melanoma progression and underscore the complexity of its underlying molecular mechanisms, offering multiple promising avenues for the development of m6A-targeted therapeutic strategies. However, a thorough examination is essential to understand the downstream effector networks linked with the identified signaling pathways. Such an analysis is crucial to determine the clinical applicability of these pathways.

### 3.4 Regulatory mechanisms and network remodeling of non-coding RNAs by METTL14

METTL14-mediated m^6^A modification is one of the core mechanisms for the precise programming of non-coding RNA (ncRNA) functions at the post-transcriptional regulatory layer. Through differential modification of distinct ncRNA classes, METTL14 profoundly modulates their processing, stability, and functional outputs, thereby reshaping the global gene regulatory network—this forms the molecular basis for its role in driving or suppressing tumor progression.

#### 3.4.1 Regulation of microRNA biogenesis

Accumulating evidence (115) has demonstrated that the METTL14 complex directly regulates the processing and maturation of primary microRNAs (pri-miRNAs) by catalyzing m^6^A modification of these transcripts. This modification is specifically recognized by the nuclear RNA-binding protein DiGeorge syndrome critical region 8 (DGCR8), a key component of the Drosha-DGCR8 microprocessor complex. The m^6^A mark enhances the affinity of DGCR8 for pri-miRNAs, thereby accelerating the processing efficiency of pri-miRNAs into precursor miRNAs (pre-miRNAs). This “METTL14-m^6^A-DGCR8” regulatory axis establishes a critical functional link between epitranscriptomic events and the miRNAome.

The biological significance of this regulatory axis has been validated across multiple pathological processes, particularly in tumorigenesis. For instance, in colorectal cancer (CRC), downregulated METTL14 expression reduces m^6^A modification levels on pri-miR-17, impairs its processing, and decreases the abundance of mature miR-17-5p. This cascade subsequently mediates chemoresistance to 5-fluorouracil (5-FU) via targeting MFN2 (103). Recent investigations in head and neck squamous cell carcinoma (HNSCC) further demonstrated that METTL3/METTL14-mediated m^6^A modification accelerates pri-miR-3690 maturation by enhancing DGCR8-dependent recognition and processing of the transcript. The upregulated miR-3690 then drives tumor cell proliferation, migration, and invasion through targeting genes including CKS2 and NKD1 (116). Collectively, these findings indicate that METTL14 precisely modulates miRNA maturation by regulating m^6^A modification of specific pri-miRNAs, thereby contributing to the control of cell fate decisions and pathological processes such as tumor progression and therapeutic resistance across diverse disease contexts.

#### 3.4.2 Bidirectional regulation of lncRNA and circRNA stability by METTL14-mediated m^6^A modification

For lncRNAs and circRNAs, m^6^A modification-dependent regulation of their homeostasis exhibits a distinct “reader-dependent” feature, which is a key determinant of their functional outcomes.

Studies have demonstrated (117) that the YTHDF2-mediated degradation pathway represents a critical regulatory direction. Upon recognition of m^6^A modifications by YTHDF2 family readers, target transcripts are typically recruited to the CCR4-NOT deadenylase complex, thereby accelerating their degradation. This mechanism may serve to eliminate tumor-suppressive ncRNAs, thus relieving their inhibitory effects on tumor progression.

Conversely, the IGF2BP-mediated stabilization pathway is equally essential. When m^6^A modifications are recognized by the IGF2BP family (IGF2BP1/2/3), RNA stability is significantly enhanced. In pancreatic cancer, METTL14 stabilizes the oncogenic lncRNA LINC00941 in an IGF2BP2-dependent manner, thereby driving the EMT process (48). In osteosarcoma, the METTL14/IGF2BP2 axis promotes tumor progression and therapeutic resistance by stabilizing the mRNA of the proto-oncogene MN1 (12). These studies uncover the mechanism by which METTL14 drives malignant tumor phenotypes through stabilizing specific oncogenic ncRNAs.

This functional antagonism, arising from the interpretation of identical m^6^A modifications by distinct readers, constitutes the molecular basis for METTL14’s context-specific regulation. The tissue-specific expression profiles of readers, their relative intracellular abundance, and the competitive dynamics of binding sites collectively determine the ultimate functional output of m^6^A modifications—this partially explains why METTL14 exhibits divergent functional modes across different tumor types.

Furthermore, m^6^A modification-dependent regulation of lncRNA/circRNA stability is also modulated by multiple factors, including RNA secondary structure, covalent modifications, and microenvironmental signals. For instance, in hepatocellular carcinoma (HCC), m^6^A-modified circSTX6 can act as a “protein sponge” to sequester the RNA-binding protein HNRNPD, thereby impairing HNRNPD’s regulatory function on its downstream target mRNAs (e.g., ATF3) (118).

#### 3.4.3 Remodeling ceRNA networks and emerging frontiers in translation regulation

m^6^A-modified lncRNAs and circRNAs are frequently activated as competing endogenous RNAs (ceRNAs), which sequester specific miRNAs to alleviate their inhibitory effects on downstream target mRNAs, thereby orchestrating extensive regulatory networks. METTL14 indirectly modulates the expression of numerous oncogenes and tumor suppressor genes by fine-tuning the abundance of these core “molecular sponges”.

In HCC, METTL14-mediated m^6^A modification facilitates the nuclear export of circFUT8. In the cytoplasm, circFUT8 acts as a miR-552-3p sponge to upregulate CHMP4B expression, thereby enhancing tumor stemness (31). In OSCC, METTL14-stabilized MALAT1 promotes tumor progression by sequestering miR-224-5p (54). In GC, METTL14 downregulates circORC5 expression via m^6^A modification; since circORC5 itself functions as a miR-30c-2-3p sponge, the tumor-suppressive activity of METTL14 is partially mediated by the circORC5/miR-30c-2-3p/AKT1S1 ceRNA axis (38). These examples underscore METTL14’s critical role as an upstream “programmer” of ceRNA networks.

Recent studies have uncovered a novel regulatory dimension: m^6^A modifications can serve as activation signals for internal ribosome entry sites (IRES), driving cap-independent translation of circRNAs containing open reading frames (ORFs) to generate biologically functional micropeptides or proteins (98, 119). This finding implies that METTL14 may regulate the production of a new class of tumor-associated functional products by modulating circRNA translation efficiency. For instance, in HCC, METTL14 upregulates circSTX6 expression in an m^6^A-dependent manner; this circRNA translates into a novel 144-amino acid polypeptide (circSTX6-144aa), which exerts independent biological functions from its parental circRNA and promotes HCC progression (118). Additionally, more complex bidirectional crosstalk between circRNAs and the m^6^A modification machinery has been revealed: in bladder cancer, circ0008399 directly binds to WTAP (a core component of the METTL14 complex), enhancing the assembly and activity of the m^6^A methyltransferase complex to regulate downstream target genes (e.g., TNFAIP3) and induce chemoresistance (120).

In summary, METTL14 constructs an intricate post-transcriptional regulatory network through multi-layered, differential m^6^A modification of non-coding RNAs. It not only precisely regulates miRNA biogenesis but also dictates lncRNA/circRNA stability via reader-dependent bidirectional mechanisms. Furthermore, by remodeling ceRNA networks and the potential translation landscape, METTL14 converts epitranscriptomic information into diverse biological outputs. This networked, context-dependent regulatory system underpins METTL14’s “dual” functions in tumors and provides a theoretical framework for ncRNA network-based precision intervention strategies.

### 3.5 The role of METTL14 in regulating the tumor immune microenvironment

The tumor immune microenvironment (TIME) is a critical niche that dictates tumor progression and therapeutic responses. Accumulating evidence indicates (121) that METTL14-mediated m^6^A modification acts as a dynamic regulator of the TIME, modulating the balance between immunosuppression and anti-tumor immunity through multi-dimensional effects on both tumor cells and infiltrating immune cells.

#### 3.5.1 Regulation of immune checkpoint expression in tumor cells

Studies have demonstrated that METTL14 directly modulates the immunogenicity of tumor cells via post-transcriptional regulatory mechanisms. A paradigmatic example is its regulation of PD-L1 expression: in glioblastoma and acute myeloid leukemia (AML), METTL14 stabilizes PD-L1 mRNA in an m^6^A-dependent manner, leading to a marked upregulation of PD-L1 protein on the tumor cell surface (17, 65). As a key immune checkpoint molecule, PD-L1 inhibits T cell activation by binding to PD-1 on T cells—a core mechanism of tumor immune evasion. This finding establishes the METTL14-m^6^A axis as a central regulator of the intrinsic immune escape program in tumors and suggests that its activity status may serve as a potential predictive biomarker for the efficacy of immune checkpoint inhibitors.

#### 3.5.2 Mediating metabolic-immune crosstalk

METTL14 also indirectly reshapes the immune properties of the TIME by driving metabolic reprogramming in tumor cells. In cervical cancer, METTL14 activates the AMPK-glycolysis pathway, resulting in extensive lactic acid accumulation in the TME (113). As a critical immunoregulatory metabolite, high lactic acid concentrations induce the polarization of tumor-associated macrophages (TAMs) toward the immunosuppressive M2 phenotype and impair the antigen-presenting function of dendritic cells (DCs), thereby attenuating anti-tumor immune responses at multiple levels.

Conversely, immune cells actively secrete signals to feedback-regulate METTL14 function in tumor cells, forming a bidirectional crosstalk network. For example, in medulloblastoma, TAM-derived exosomes downregulate METTL14 expression in tumor cells (68). This downregulation reduces global m^6^A modification levels, relieving transcriptional repression of the transcription factor FOXD1. Upregulated FOXD1 then inhibits the secretion of key chemokines, suppressing CD8^+^ T cell infiltration into the tumor site and ultimately shaping an “immune-excluded” microenvironment. This mechanism reveals that immune cells in the TIME can “hijack” the epitranscriptomic program of tumor cells via exosomes to actively construct a niche conducive to immune escape.

Additionally, METTL14 may indirectly influence immune cell function by regulating broader metabolic pathways (e.g., lipid metabolism). In nasopharyngeal carcinoma, for instance, METTL14 modulates acetyl-CoA metabolism and histone acetylation via ANKRD22, forming a positive feedback loop (51). These findings suggest that the METTL14-mediated metabolic-immune regulatory network is highly complex and context-dependent, constituting a multi-dimensional metabolic basis for tumor immune escape.

#### 3.5.3 Direct regulation of immune cell function

METTL14’s functions extend directly to tumor-infiltrating immune cells, influencing their differentiation and functional states. In colorectal cancer (CRC) TAMs, reduced METTL14 expression decreases m^6^A-dependent degradation of the immunosuppressive factor EBI3 mRNA, leading to EBI3 protein accumulation and direct inhibition of effector T cell function (14). This mechanism underscores the complexity of METTL14 in promoting immune tolerance through target-specific regulation across distinct cellular compartments.

Notably, METTL14’s immunoregulatory functions exhibit marked context dependence. For example, in rectal cancer, high METTL14 expression in tumor cells correlates with enhanced immune cell infiltration and improved prognosis (35), indicating that it may exert distinct immunoregulatory properties in specific tissue or molecular contexts—further highlighting the high complexity of its functions.

In summary, METTL14 reshapes the immunosuppressive TIME through three core mechanisms: direct upregulation of immune checkpoints in tumor cells; metabolic reprogramming to alter TME physicochemical properties; and direct modulation of immune cell function. This provides a novel epitranscriptomic perspective for understanding immune escape and lays a theoretical foundation for developing m^6^A-targeted combinatorial immunotherapies. Future studies should further elucidate the heterogeneous patterns of its functions to advance precision immunotherapy.

## 4 Conclusion and perspectives

**Figure 3:**
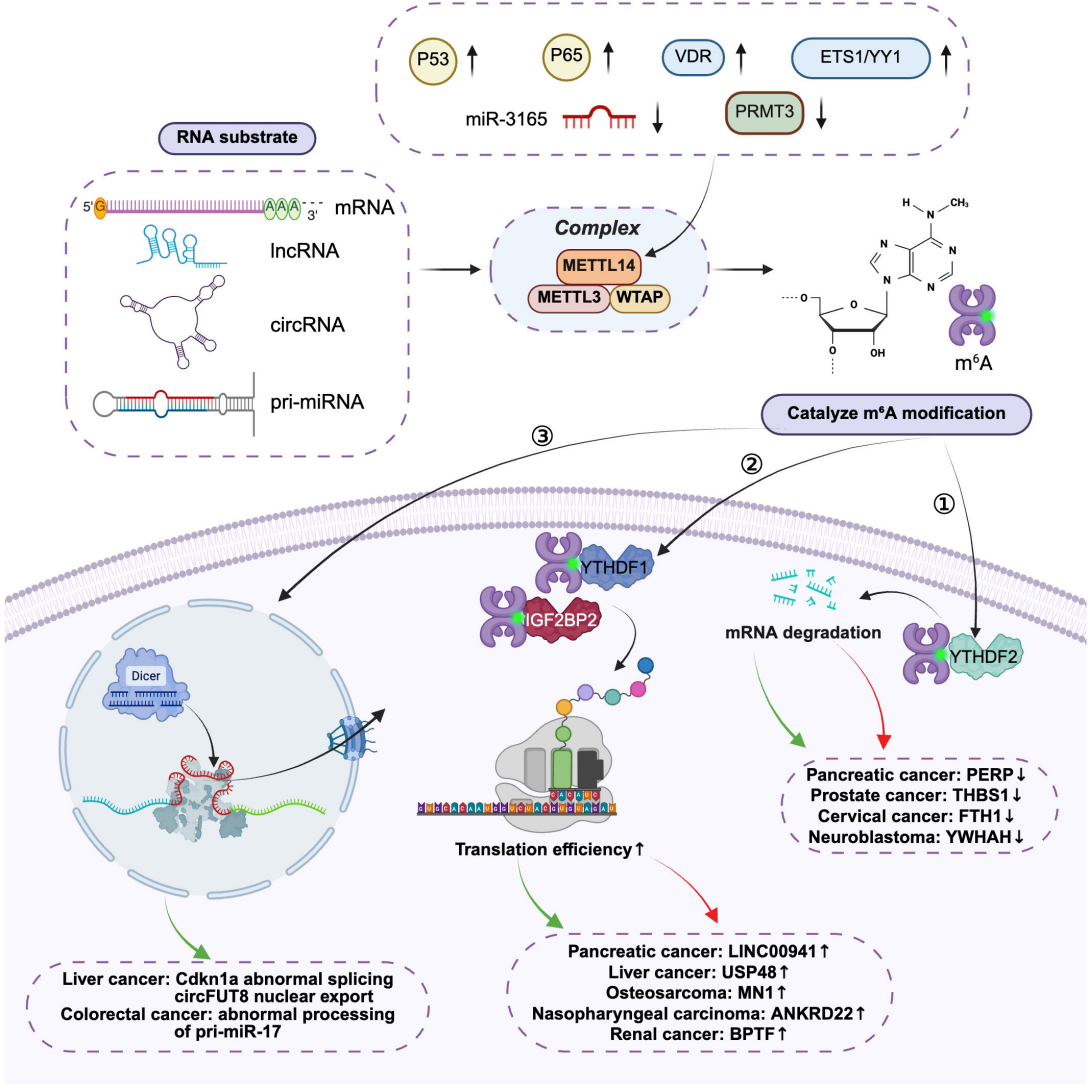
Dual functionality and clinical relevance of the METTL14-m^6^A axis in tumors are context-dependent: This figure depicts RNA substrates, the involvement of the METTL14/METTL3/WTAP complex, catalytic processes, mRNA fate (degradation and translation), and associations with cancers including hepatocellular carcinoma, pancreatic cancer, and colorectal cancer—annotated with affected genes and corresponding biological outcomes.

This This article systematically reviews the functional duality of METTL14 across diverse tumor types, clarifying its role as a core component of the m^6^A methyltransferase complex that exerts context-dependent, multi-faceted regulatory effects in distinct malignancies. Its oncogenic or tumor-suppressive activities are dynamically governed by multi-layered factors including tissue origin, molecular subtypes, genetic background, and the TME (6–8, 35, 85, 86, 97), highlighting the precise plasticity of m^6^A modification in tumor progression. On this basis, **Figure 3** integrates the regulatory pathways of the METTL14-m^6^A axis across different tumor types via a multi-tiered network, visually depicting its functional duality and clinical relevance using green arrows (tumor-suppressive effects) and red arrows (oncogenic effects).

From a molecular mechanistic perspective, while METTL14’s functional direction diverges across tumors, its actions exhibit striking commonalities. First, key signaling pathways such as PI3K/AKT and Wnt/β-catenin serve as recurrent regulatory hubs for the METTL14-m^6^A axis (33, 40, 62, 96); by modifying pathway components (e.g., PTEN, SOX4, β-catenin, c-Myc), METTL14 selectively activates or represses these pathways in tumor-specific contexts, thereby driving or inhibiting tumor growth (7, 12, 33). Second, METTL14 is broadly involved in cell cycle regulation and apoptosis, directly governing tumor cell proliferation and survival via m^6^A modification of critical molecules like Cyclin D1 (29, 71). In immune regulation, METTL14 follows a consistent paradigm: it upregulates immune checkpoint molecules (e.g., PD-L1) (17, 65) while synergistically shaping immunosuppressive microenvironments through metabolic reprogramming or modulation of immune cell functions (e.g., EBI3 in tumor-associated macrophages (TAMs)) (14, 113). Additionally, as a master regulator of nncRNA networks, METTL14 broadly modulates ceRNA networks and downstream signaling by influencing pri-miRNA processing and the stability of lncRNAs/circRNAs (31, 38, 48, 54, 103, 116). These recurring downstream targets and pathways underscore a core mechanistic framework for METTL14 function—while its effect direction varies with tumor context, its molecular “handles” converge on regulating growth signals, cell cycle, immune escape, and RNA networks, which are pivotal nodes in tumor biology. Notably, its functional duality stems primarily from the selective targeting of downstream substrates and differential decoding by m^6^A reader proteins (e.g., degradation-promoting YTHDF2 vs. stabilization-promoting IGF2BPs) (10, 12, 48, 78), enabling identical modifications to elicit diametrically opposed biological outcomes in distinct cellular environments.

Looking ahead, research must transition to more refined, dynamic paradigms to dissect the critical thresholds and mechanisms underlying METTL14’s functional transitions. It is recommended to integrate single-cell multi-omics and spatial transcriptomics to map its dynamic regulatory networks within specific molecular subtypes and TME contexts. Concurrently, translating METTL14 and its associated molecular markers into precise tumor stratification—particularly for predicting responses to chemotherapy, targeted therapy, and immunotherapy—holds significant clinical value.

In terms of clinical translation, strategies targeting the METTL14-m^6^A axis require balancing high specificity and context dependency. For tumor subsets definitively driven by this axis, developing METTL14-selective inhibitors or targeting its key downstream effectors (e.g., FOXD1) shows promise (15, 68). More compelling is the design of combinatorial therapies, such as combining METTL14 inhibitors with PI3K/AKT pathway inhibitors or PD-1/PD-L1 blockers, to overcome bypass activation and synergistically enhance anti-tumor immunity (17, 68). Additionally, exploring synthetic lethal interactors of METTL14 and emerging targets like METTL14-mediated circRNA translation products offers novel avenues for tumor therapy (98, 118, 119).

In conclusion, in-depth research on METTL14 has transcended the traditional “oncogene/tumor suppressor” dichotomy, framing it as a precise, highly context-dependent regulatory hub. By systematically dissecting its molecular networks in specific tumor contexts and developing tailored diagnostic and combinatorial therapeutic strategies, we aim to provide new scientific foundations and clinical pathways for overcoming tumor heterogeneity and treatment resistance.
